# Matrix metalloproteinase-9 increases and Interleukin-10 reduces with increase in body mass index in polycystic ovary syndrome: A cross-sectional study

**DOI:** 10.18502/ijrm.v13i8.7502

**Published:** 2020-08-19

**Authors:** Angel Mercy Sylus, Hanumanthappa Nandeesha, Thiagaraju Chitra

**Affiliations:** ^1^Department of Biochemistry, Jawaharlal Institute of Post Graduate Medical Education and Research, Puducherry, India.; ^2^Obstetrics and Gynecology, Jawaharlal Institute of Post Graduate Medical Education and Research, Puducherry, India.

**Keywords:** Body mass index, Interleukin-10, Nitric oxide, Matrix metalloproteinase-9.

## Abstract

**Background:**

Obesity, inflammation and alterations in matrix metalloproteinase-9 (MMP-9) and nitric oxide (NO) levels are involved in the development of polycystic ovary syndrome (PCOS).

**Objective:**

To investigate the relationship of MMP-9, NO and interleukin-10 (IL-10) with the increase in body mass index (BMI) in women with PCOS.

**Materials and Methods:**

Sixty two infertile PCOS women were included in the study. Serum levels of NO, IL-10 and MMP-9 were assessed in the women with increase in BMI.

**Results:**

MMP-9 was significantly increased (p = 0.029) and IL-10 (p = 0.015) was significantly reduced in obese PCOS subjects compared to those with lesser BMI. MMP-9 levels positively correlated with the duration of infertility (r = 0.253, p = 0.047) and negatively correlated with NO levels (r = - 0.259, p = 0.042). A significant negative correlation between the interleukin-10 levels and the BMI (r = - 0.272, p = 0.033) was also found in the PCOS subjects.

**Conclusion:**

MMP-9 levels are increased in obese PCOS women and it is associated with NO levels and the duration of infertility.

## 1. Introduction

Polycystic ovary syndrome (PCOS) is a common multifaceted hormonal disorder that affects almost 5-10 % of women in the reproductive age group (1). Reproductive abnormalities like ovulatory dysfunction, infertility and features of hyperandrogenism are the most common manifestations. PCOS subjects are at an increased risk of developing metabolic complications like diabetes mellitus, and cardiovascular disease (2).

Several investigators have emphasized the role of obesity as a contributing factor in the pathogenesis of PCOS. Body mass index (BMI) was found to be increased in 50-60% of women with PCOS (3). Obesity is known to be associated with infertility and the influence of obesity on the exacerbation of clinical features and hormonal disturbances of PCOS has been revealed the past by many earlier studies (4). Healthy dietary habits and weight reduction have shown a beneficial impact on the metabolic state, hyperandrogenemia, ovulatory function and pregnancy rates in PCOS suggesting the importance of maintaining a healthy body weight for women with PCOS (5).

Matrix metalloproteinase-9 (MMP-9) is a zinc-dependent enzyme involved in the tissue remodeling of the ovary and uterus, which is responsible for growth of the follicle, formation of corpus luteum and blastocyst implantation (6). Elevated MMP-9 expression has been reported to be involved in trophoblast invasion during pregnancy (7). Previous studies have hypothesized that increased MMP-9 levels might be related to menstrual irregularities and increased risk of cardiovascular disease in PCOS patients (6, 8).

Nitric oxide (NO) plays a crucial role in pregnancy and it promotes embryonic development, maintains the fetoplacental unit and sustains pregnancy (9). Reduced NO levels in women with PCOS were found to be related with spontaneous pregnancy (10).

Inflammation is usually associated with PCOS, which influences ovarian folliculogenesis, altered steroidogenesis in ovary and hyperinsulinemia in these women (11). Interleukin-10 (IL-10), an anti-inflammatory cytokine regulates the action of pro-inflammatory cytokines during inflammation. IL-10 gene polymorphism has been reported in PCOS and reduced IL-10 levels have been documented in women with PCOS (11, 12).

Since obesity and alteration in inflammation and matrix metalloproteinases are important underlying factors associated with the metabolic, hormonal and reproductive abnormalities in PCOS (13-15), this study was intended to determine the influence of increase in body mass index on MMP-9, nitric oxide and interleukin-10 in PCOS.

## 2. Materials and Methods

### Subjects

The present study was a cross sectional study conducted in the department of Biochemistry and Obstetrics & Gynaecology, Jawaharlal Institute of Post Graduate Medical Education and Research, Puducherry, India from December 2014 to July 2016. Sixty two PCOS subjects in the age group of 20-35 yr, were enrolled in the study. The diagnosis of PCOS in the participants was made based on the revised Rotterdam criteria 2003 (16). The subjects with medical comorbidities, fallopian tube disorders, ovarian failure, endometriosis and those who were using metformin were excluded from the study.

### Clinical evaluation

The study subjects were evaluated by a detailed clinical history, treatment history and physical examination.

### Sample collection

Venous blood samples of 5 ml were collected from the participants. Serum was separated and stored at - 80°C and used to assess the NO, IL-10 and MMP-9 levels.

### Biochemical analysis

Colorimetric method (Oxford Biomedical Research, USA) was used to analyze serum NO levels. Serum IL-10 (Diaclone, France) and MMP-9 (Raybiotech, Mexico) were analyzed by ELISA.

### Ethical consideration

The present study was approved by the Institution ethics Committee for human studies (Ref: JIP/IEC/2014/8/367). Written informed consent was obtained from all the subjects prior to the study.

### Statistical analysis

All continuous data are displayed as mean ± SD. One way ANOVA was used to compare the parameters between the three groups of PCOS. Correlations were determined using Pearson correlation analysis. A p-value < 0.05 was considered as significant. Statistical analysis was done using IBM SPSS Statistics 16 (IBM Corporation, Somers, NY).

## 3. Results

The differences in age, clinical characteristics and biochemical parameters among the PCOS subjects, grouped on the basis of their BMI, are shown in Table I. PCOS subjects with BMI > 30 kg/m${}^{2}$ had significantly higher levels of MMP-9 (p = 0.029) compared to those with BMI between 25 and 28 kg/m${}^{2}$. IL-10 was found to be significantly lower in PCOS subjects with BMI between 25 and 28 kg/m${}^{2}$ (p = 0.002), BMI between 28 and 30 kg/m${}^{2}$ (p = 0.002) and BMI > 30 kg/m${}^{2}$ (p = 0.015) compared to those with a normal BMI < 25 kg/m${}^{2}$. There was no significant difference in the no levels, age and duration of infertility among the PCOS subjects.

Table II shows the correlation of MMP-9 with age, BMI and biochemical parameters like IL-10 and NO in the PCOS subjects. MMP-9 was positively correlated with the duration of infertility (r = 0.253, p = 0.047) and negatively correlated with NO (r = -0.259, p = 0.042). IL-10 was negatively associated with BMI (r = -0.272, p = 0.033) and the duration of infertility was positively associated with age (r = 0.374, p = 0.003) in PCOS subjects.

**Table 1:** Mean and SD of age, clinical characteristics and biochemical parameters in PCOS subjects (n = 62) with increase in BMI


**Parameters**	**PCOS subjects (n = 62)**			
	**BMI < 25 kg/m${}^{2}$ (n = 14)**	**BMI 25-28 kg/m${}^{2}$ (n = 32)**	**BMI 28-30 kg/m${}^{2}$ (n = 9)**	**BMI > 30 kg/m${}^{2}$ (n = 7)**
**Age**	24.8 ± 2.5	25.4 ± 2.8	26.1± 3.5	24.7 ± 2.3
**Duration of infertility**	2.5 ± 0.73	2.54 ± 0.62	3.05 ± 1.28	2.85 ± 0.69
**MMP-9**	2995 ± 561	2691 ± 692	3171 ± 673	3319 ± 766*
**Interleukin-10**	10.96 ± 8.11	5.42 ± 4.25*	3.68 ± 3.0*	4.89 ± 3.95*
**Nitric oxide**	20.68 ± 8.36	18.53 ± 5.96	19.2 ± 5.35	18.2 ± 4.43
*P < 0.01 compared to PCOS subjects with BMI < 25 kg/m${}^{2}$, **p < 0.05 compared to PCOS subjects with BMI 25 - 28 kg/m${}^{2}$ PCOS subjects with BMI > 30 kg/m${}^{2}$ had significantly higher levels of MMP-9 (p < 0.05) compared to those with BMI between 25 and 28 kg/m${}^{2}$. Interleukin-10 was found to be significantly lower in PCOS subjects with BMI between 25 and 28 kg/m${}^{2}$ (p < 0.01), BMI between 25 and 28 kg/m${}^{2}$ (p < 0.01) and BMI > 30 kg/m${}^{2}$ (p < 0.01) compared to those with a normal BMI < 25 kg/m${}^{2}$. There was no significant difference in the nitric oxide levels, age and duration of infertility among the PCOS subjects. Statistical test used: One-way ANOVA was used to compare the parameters between the three groups of PCOS				

**Table 2:** Correlation of MMP-9 with age, BMI, and biochemical parameters in PCOS subjects (n = 62)


**Parameter**	**r**	**P-value**
**Age**	0.073	0.574
**BMI**	0.165	0.199
**Nitric oxide**	-0.259	0.042
**Interleukin-10**	0.285	0.025
**Duration of infertility**	0.253	0.047
MMP-9 was positively correlated with the duration of infertility (r = 0.253, p = 0.047) and negatively correlated with nitric oxide (r = - 0.259, p = 0.042) in PCOS. Statistical test used: Pearson correlation analysis		

## 4. Discussion

In the present study, MMP-9 was increased and IL-10 was reduced in PCOS women with increase in BMI. MMP-9 was associated with NO and duration of infertility in PCOS.

Several factors such as inflammation, endothelial dysfunction and altered ovarian remodelling are known to play a role in the pathogenesis of PCOS and its complications including infertility (17-19). Obesity has got a major influence on the clinical outcome of PCOS as evident by the presence of hyperandrogenism, insulin resistance, decreased ovulation as well as lower pregnancy rates in PCOS women with obesity in comparison with non-obese PCOS women (20).

MMP-9, a matrix metalloproteinase produced by the ovary is involved in various phases of female reproduction. High MMP-9 levels in PCOS adversely affects ovulation and fertility, as it alters the extracellular matrix remodelling thus causing aberrant follicular atresia and increased ovarian stromal tissue (21). MMP-9 is reported to be strongly associated with obesity and furthermore the presence of a high BMI has also been hypothesized to promote poor ovulation and thereby lesser chances of pregnancy (22). In our study, we observed that MMP-9 levels were higher in infertile women with PCOS having a BMI > 30 kg/m${}^{2}$ compared to those with BMI between 25 and 28 kg/m${}^{2}$. These findings were supported by previous studies that have reported high MMP-9 in obese subjects (22, 23). As body weight increases, fat cells are enlarged and adipose tissue is expanded and differentiated. During differentiation of adipose tissue, the activity of MMP-9 is induced resulting in remodeling of stromal matrix (24).

NO has a pivotal role in endothelial function by which it regulates the uterine blood flow, relaxes the uterine myometrium and reduces the feto-placental vascular resistance during pregnancy (25) NO levels are found to be reduced both in obesity and PCOS subjects which might be associated with adverse pregnancy outcome (26, 27). In our study, we observed a reduction in the nitric oxide levels as BMI increased however, it was not statistically significant. Decreased serum NO levels in PCOS have been attributed to elevated asymmetrical dimethyl arginine (ADMA) in PCOS subjects associated with hyperandrogenemia and insulin resistance (28, 29).

Extensive studies have documented the role of inflammation in PCOS and reported an imbalance in the levels of pro and anti-inflammatory cytokines in these subjects (11). Obesity and inflammation are known to occur concurrently (30). The presence of hyperandrogenemia in PCOS stimulates adipocyte hypertrophy and hypoperfusion, release of inflammatory mediators and development of chronic inflammation, consequently resulting in insulin resistance and cardiovascular complications (28). Low levels of IL-10 were observed in PCOS and it was found to affect the pregnancy rates and outcomes (31). Our data demonstrated a significant reduction in the serum IL-10 levels of infertile women with PCOS who had a BMI in the range of 25-28 kg/m${}^{2}$, 28-30 kg/m${}^{2}$, and > 28 kg/m${}^{2}$ compared to those who had a normal BMI. Also IL-10 was negatively associated with BMI suggesting that as BMI increases, IL-10 reduces in PCOS subjects. These findings were in agreement with previous reports that demonstrate reduction in IL-10 levels in obesity and PCOS (31, 32). Since weight gain is associated with inflammation (33), there is reduction of anti-inflammatory cytokines like IL-10.

In the present study, MMP-9 levels negatively correlated with NO levels and positively correlated with the duration of infertility in PCOS subjects. These findings suggest that as the duration of infertility increases, the MMP-9 level increases which in turn reduces NO levels leading to the complications of PCOS.

In the present study we have investigated whether these markers are altered in PCOS women with increase in body weight / BMI. Alteration in these markers can lead to reduction in ovulation. Based on the findings of our study we suggest that weight reduction in PCOS can reduce MMP-9 and increase IL-10, which may improve ovulation in these subjects. The main limitations of the study are the small sample size and non-inclusion of healthy control groups. Moreover we did not estimate other inflammatory cytokines due to financial constraints.

## 5. Conclusion

The present study concludes that as BMI increases MMP-9 is increased and IL-10 is reduced in PCOS subjects. The association of MMP-9 with NO and duration of infertility indicates that high MMP-9 levels may cause endothelial dysfunction which might be responsible for complications in PCOS subjects with obesity. Further studies are required to investigate whether weight reduction reduces MMP-9 levels and the complications of PCOS in obese subjects.

##  Conflict of Interest

The authors report no conflict of interest.
